# Optimising reflux symptom burden and patient compliance during PPI washout periods for oesophageal pH monitoring studies: should we be more proactive with alginate use?

**DOI:** 10.1136/bmjgast-2022-001078

**Published:** 2023-02-06

**Authors:** Sophia Agwaonye, Dipesh H Vasant

**Affiliations:** 1 Neurogastroenterology Unit, Wythenshawe Hospital, Manchester University NHS Foundation Trust, Manchester, UK; 2 Division of Diabetes, Endocrinology and Gastroenterology, University of Manchester, Manchester, UK

**Keywords:** OESOPHAGEAL pH MONITORING, OESOPHAGEAL PHYSIOLOGY, OESOPHAGEAL REFLUX

In this edition of *BMJ Open Gastroenterology,* an important randomised clinical trial by Vales *et al* has investigated the structured use of alginates during preinvestigation proton pump inhibitor (PPI) washout to aid compliance and alleviate symptom burden.^1^

Under normal circumstances, 24-hour oesophageal pH/impedance monitoring requires patients to stop taking their PPI at least a week prior to the investigation in order to accurately measure their normal acid levels.^2^ Unfortunately, it is often difficult for patients to abstain from PPIs for fear of exacerbating their reflux symptoms, which is known to occur due to rebound acid hypersecretion after PPI withdrawal.^2^ The worsening of symptoms can lead to failure to attend appointments, or surreptitiously taking PPIs during the period of abstention, which is detrimental to the sensitivity of the investigation.

Alginates are natural products derived from seaweed, which jellifies to form a raft coating property, that act as a physical barrier to acid reflux. Importantly, as alginates do not affect production of acid, they can be ingested up to the night before the test.^1^ In the Vales *et al* clinical trial, 60 participants were investigated with oesophageal 24-hour pH/impedance testing having been established on PPI medication for 4 weeks or more. PPIs and H2 receptor antagonists were stopped a week prior to the investigation; however, antacids and alginates could be used up to the night before.^1^ Interestingly, while those who were assigned to a control group experienced a significant exacerbation of symptoms after stopping PPI therapy, those who received four daily doses of Gaviscon Advance (treatment group) had no significant increase in symptoms.^1^ These findings suggest that a proactive introduction of alginates during preinvestigation PPI washout periods can be successful in alleviating patient reflux symptoms.^1^

The main limitation of this trial is that the baseline Gastroesophageal Reflux Disease-Health Related Quality of Life scores were different in the two groups, with a higher symptom severity and reflux burden in those that were in the structured alginate (treatment) group. While this imbalance may have been due to the small sample size or lack of diversity in the study, it could also be considered a strength of the study as it confirms that patients who were more likely to have had a symptom deterioration after stopping PPI, those with higher baseline symptom severity and objectively more severe gastro-oesophageal reflux, benefitted from a regular alginate use before their test. Another limitation is that the open-label nature of the study left room for bias. However, this effect was minimised because patients from the control group were allowed to take alginates whenever they felt was necessary, which represented a ‘real-world’ scenario, as opposed to the treatment group, who used the alginate in a regular, structured fashion, as per protocol.

The findings of this trial confirm that alginate use prior to oesophageal pH testing is effective in preventing symptom deterioration and support the use of alginates in a more structured way during weeklong PPI washout periods prior to acid reflux studies. These data are important for several reasons. First, the cost of implementation for patients and healthcare providers in routine practice is relatively low when compared with the costs of the investigation. Second, effective strategies such as this to prevent symptom deterioration are likely to improve compliance with PPI cessation and attendance at gastrointestinal physiology centres. Following the COVID-19 pandemic, there has been a consequent increase in the waiting time for oesophageal reflux testing, and therefore, there is a need to optimise compliance and reduce non-attendance rates.^3^

In a clinical setting, where patients do not attend their allocated appointment or are not fully compliant with preinvestigation PPI washout due to inability to cope with their worsening symptoms, the patient’s appointment may ultimately need to be rebooked for the next available allocation (figure 1). This process would therefore be a loss of opportunity for other patients on the waiting list to be seen and would also have further knock-on effects on waiting times. Therefore, the importance of using alginates during preinvestigation PPI washout could be quite important, given the potential to reduce non-attendance and waiting times while alleviating patients’ symptoms and aiding compliance during PPI washout. In figure 1, we suggest a potential workflow where structured alginate use could be integrated into routine practice to optimise the utility of testing.

**Figure 1:**
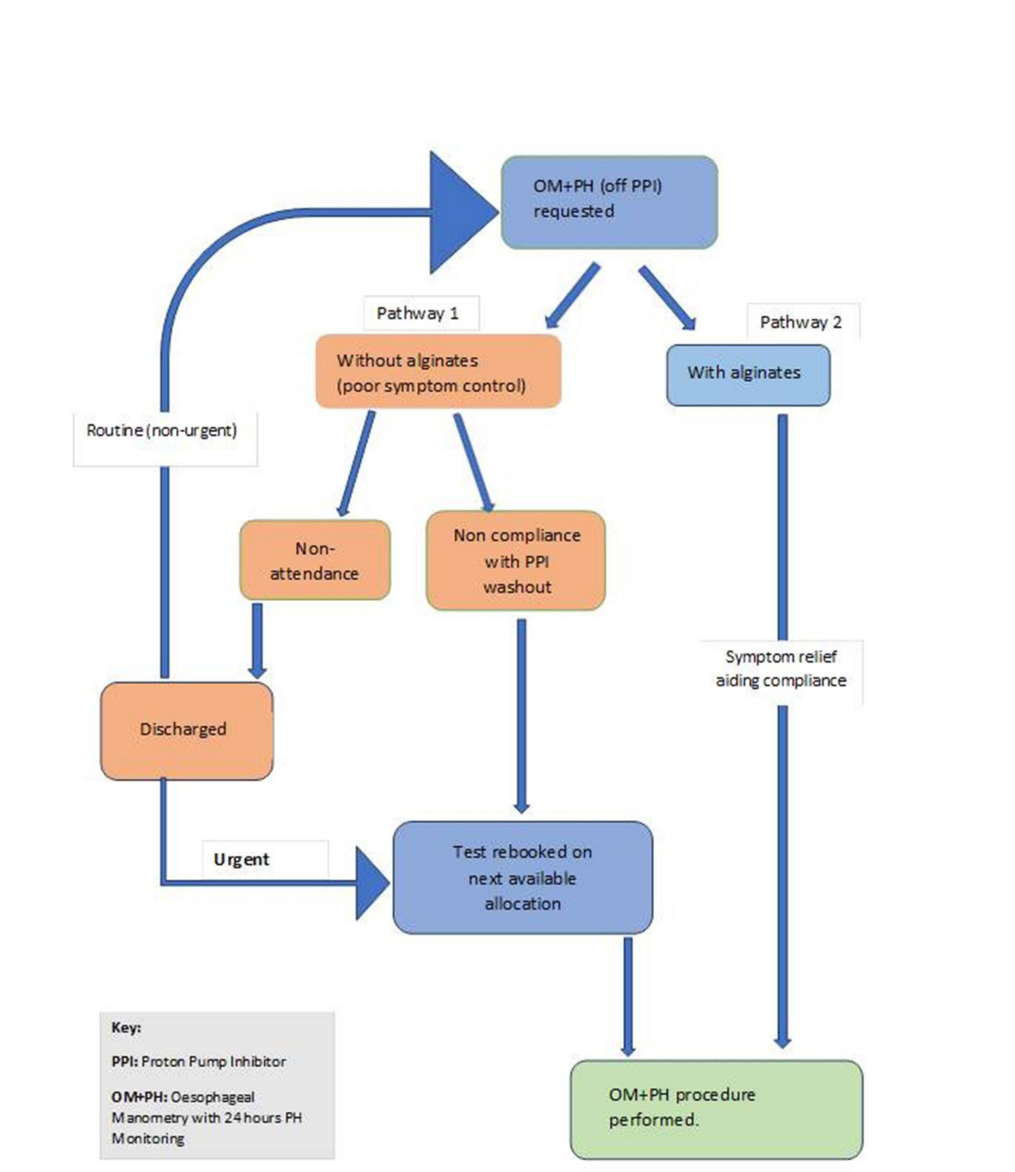
Schematic illustration of the potential benefits of introducing structured alginate use in routine practice during PPI washout before 24-hour oesophageal pH studies. OM+pH, oesophageal manometry with 24-hour pH monitoring; PPI, proton pump inhibitor.

Finally, the optimal use of oesophageal reflux testing is entirely dependent on the context and clinical questions being asked. Therefore, the use of structured alginate use as a means to enhancing compliance during PPI washout could prevent an inappropriate ‘on PPI’ study which may become difficult to interpret and might even prevent the relevant clinical question being answered. With advances in technologies over the past decade, 24-hour oesophageal pH impendence studies are now used not only to quantify the burden of gastro-oesophageal reflux disease and guide the need for antireflux surgery in refractory cases but also to enable us to understand the mechanisms at play in symptomatic individuals and clinically phenotype patients. This has enabled delivery of a more personalised approach to caring for patients with reflux symptoms in order to tailor therapies. Such has been the progress in this field that the symptomatic patient can now be separated into those with symptoms that are driven by acid and those that are not. Therefore, an inappropriate on PPI study would be a missed opportunity to differentiate between these subgroups. There is now a positive move away from the dated ‘ladder-based approach’ to escalating reflux treatment in patients with refractory symptoms to a more considered, holistic, biopsychosocial approach, considering a multidimensional clinical profile including results from oesophageal reflux studies.^4^ In this context, oesophageal pH/impedance testing has informed the Rome IV diagnostic classification for patients with non-acid-driven symptoms.^5^ This has led to the separation of those with truly PPI refractory reflux, PPI dependent reflux and pathological reflux from those with non-acid reflux and those with oesophageal reflux hypersensitivity who have normal acid exposure (‘off PPI’) but have a positive symptom correlation to trivial amounts of acid, and those with functional heartburn who have no positive symptom correlation and no evidence of pathological reflux.^5^

Structured alginate use during PPI washout therefore has the potential to improve the accuracy of oesophageal reflux testing and influence the accurate diagnosis and management of a patient. If interpreted correctly, in the right context, with compliance during PPI washout, oesophageal reflux testing can prevent futile PPI escalation and antireflux surgeries in those who do not have acid-driven symptomatology and could potentially open doors for alternative effective treatments such as neuromodulators and brain–gut behavioural therapies.

Overall, the study by Vales *et al* answers an important clinical question and is likely to inform future practice in the field of oesophageal physiology, with the potential to improve the quality-of-life and outcomes for thousands of patients with reflux undergoing acid reflux investigations, and does therefore suggest that we should be more proactive with the use of alginates during PPI washout periods.

## Data Availability

No data are available.
